# Supplementary material for: Enhanced Expression of ARK5 in Hepatic Stellate Cell and Hepatocyte Synergistically Promote Liver Fibrosis
Source: Int J Mol Sci. 2022 Oct 28;23(21):13084. doi: 10.3390/ijms232113084 (PMC9655442; doi:10.3390/ijms232113084)
Supplement: Supplementary file 1 [file ijms-23-13084-s001.zip › ijms-1938133-supplementary.pdf]

Supplementary Material

Supplementary Figures

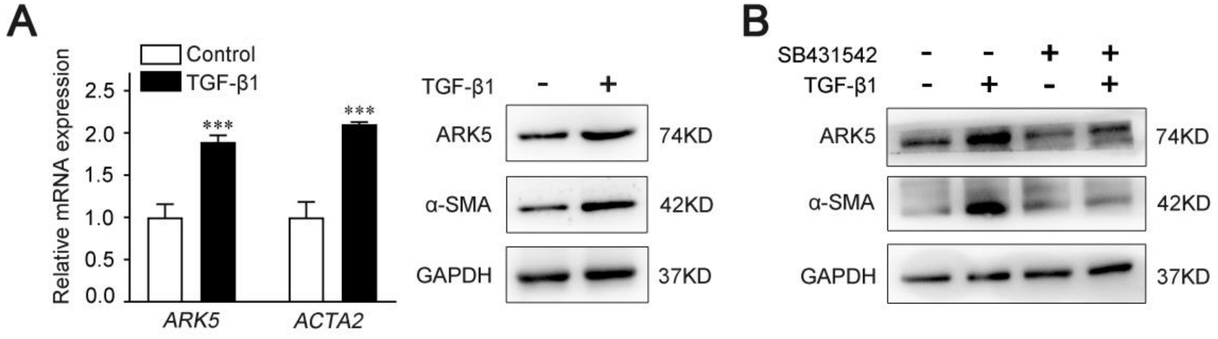

Figure S1. TGF-β1 induces increased ARK5 expression in LX2. (**A**) qPCR and Western blot for ARK5 and α-SMA in LX2 treated with or without TGF-β1 for 48 h. (**B**) Western blot for ARK5 and α-SMA in LX2 cells in indicated conditions. Cells were treated with SB431542 (10 mM), TGF-β1 (10 ng/ml) or SB431542 plus TGF-β1 for 48 h. *** $p < 0.001$ versus Control group.

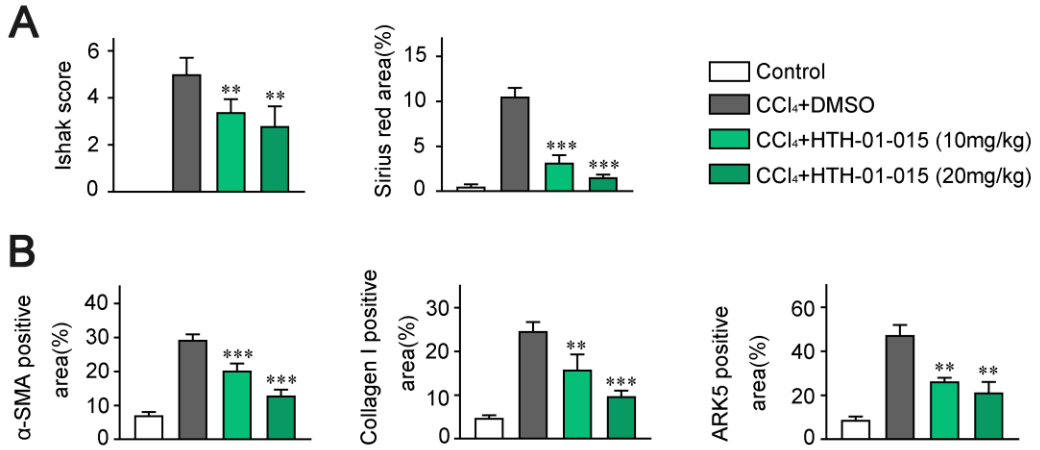

Figure S2. Histological statistical analysis of the treatment effect of HTH-01-15. (**A**) HTH-01-015 reduced Ishak fibrosis score and Sirius red staining area. (**B**) HTH-01-015 reduced the immunohistochemical staining area of α-SMA and Collagen I and ARK5. Mean ± SD (n = 3 per group). ** $p < 0.01$, and *** $p < 0.001$ versus CCl_4 + DMSO group.
